# Causal Relationships Between Plasma Metabolites, Inflammatory Factors, and Oral Cancer Risk: A Comprehensive Mendelian Randomization Study With Mediation Analysis

**DOI:** 10.1155/humu/4387817

**Published:** 2026-02-06

**Authors:** Shaonan Hu, Chufeng Liu

**Affiliations:** ^1^ Stomatological Hospital, School of Stomatology, Southern Medical University, Guangzhou City, Guangdong Province, China, fimmu.com

## Abstract

This study conducted a large‐scale Mendelian randomization analysis using genome‐wide single nucleotide polymorphisms (SNPs) as instrumental variables to investigate the causal relationships between 1400 circulating metabolites and oral cancer risk. The genetic data were derived from the Canadian Longitudinal Study on Aging (CLSA) cohort and the IEU OpenGWAS database. The study employed germline genetic variants captured in genome‐wide association studies for causal inference, combined with mediation analysis and CAL‐27 cell experimental validation. The results identified 61 metabolites with significant causal relationships with oral cancer through SNP instrumental variables (29 with protective effects and 32 increasing risk) and revealed 14 inflammatory factors as key mediating variables, with mediation effects accounting for 1.4%–17.4% of the total effects. Cell experiments further confirmed that aspartate significantly downregulates CCL11 expression and secretion and exerts anti‐inflammatory effects by suppressing inflammatory factors, including IL‐1*β*, IL‐6, and TNF‐*α*. Conversely, CCL11 overexpression promotes malignant cellular behavior, but these effects can be reversed by aspartate through inhibition of NF‐*κ*B and MAPK signaling pathways. This study elucidates a genetic variant‐driven “metabolism‐inflammation” carcinogenic pathway, providing novel insights into the mechanisms of oral cancer development and demonstrating significant translational potential for precision prevention and precision therapy.

## 1. Introduction

Oral cancer represents the sixth most common malignancy worldwide and constitutes a major public health concern, with approximately 405,000 new cases and 211,000 deaths reported annually [1]. Despite significant advances in treatment modalities, the prognosis for oral cancer patients remains poor, with 5‐year survival rates below 50% in most countries due to late‐stage diagnosis and limited effective therapeutic options [2]. The epidemiological landscape demonstrates considerable global variation, with the highest incidence rates observed in Melanesia, South‐Central Asia, and Eastern Europe [3]. Recent studies have revealed an alarming trend of increasing incidence among younger populations who lack traditional risk factor exposures [4]. Contemporary epidemiological data demonstrate a significant demographic shift in oral squamous cell carcinoma, with cases in patients under 40 years increasing from 3%–5% in the 1970s–1980s to approximately 10% currently [5], including fourfold increases in specific younger cohorts and notable rises among women aged 18–44 years lacking traditional risk factor exposures [6]. Metabolomics has emerged as a powerful tool for cancer biomarker discovery, offering insights into downstream effects of genetic and environmental perturbations on cellular metabolism [7]. Recent advances have identified distinct metabolic signatures in various cancers, with plasma metabolomics proving particularly valuable due to its noninvasive nature [8]. Chronic inflammation plays a pivotal role in oral carcinogenesis, with approximately 15% of cancers attributable to inflammatory processes [9]. Salivary cytokines, including IL‐8, IL‐6, TNF‐*α*, and IL‐1*β*, have emerged as promising noninvasive biomarkers for oral cancer detection, with TNF‐*α* demonstrating the highest diagnostic accuracy (sensitivity: 79%, specificity: 92%), followed by IL‐6 (sensitivity: 75%, specificity: 86%), IL‐8 (sensitivity: 80%, specificity: 80%), and IL‐1*β* (sensitivity: 66%, specificity: 75%) [10].

Mendelian randomization (MR) has gained prominence as a powerful epidemiological method for inferring causal relationships by utilizing genetic variants as instrumental variables, minimizing confounding and reverse causation biases [11]. The ability of MR to minimize confounding and reverse causation biases stems from the random allocation of genetic variants at meiosis, which creates natural randomization analogous to randomized controlled trials [12]. Seminal methodological reviews have established that genetic variants, being fixed at conception and preceding disease onset, are inherently protected from reverse causation and largely independent of environmental and lifestyle confounders that typically plague observational studies [13–15]. This fundamental advantage has been extensively validated across diverse epidemiological applications, demonstrating MR′s superior capacity to overcome the limitations of conventional observational research in establishing causal inference [16]. Recent applications have demonstrated causal associations between lifestyle factors and various cancer types, including sedentary behavior and pancreatic cancer risk [17].

Despite significant advances in understanding oral cancer pathogenesis, several critical knowledge gaps persist that limit our ability to develop effective prevention and treatment strategies. While metabolomic studies have been extensively conducted in breast, lung, and colorectal cancers, oral cancer presents a unique metabolic environment characterized by direct exposure to salivary metabolites and the oral microbiome, making metabolomic investigations particularly valuable for understanding disease mechanisms [18]. The oral cavity′s distinctive anatomical location allows for noninvasive sampling of saliva, which contains metabolites that directly reflect local pathological processes and may serve as accessible biomarkers for early detection [19]. However, comprehensive analyses examining causal relationships between circulating metabolites and oral cancer risk remain limited [20]. Although inflammatory factors are recognized as important contributors to oral carcinogenesis, the mechanistic pathways through which specific inflammatory mediators influence oral cancer development remain poorly characterized [21]. Understanding the complex interplay between metabolic perturbations and inflammatory responses in cancer development requires sophisticated analytical approaches that can disentangle direct effects from those mediated through intermediate biological pathways. Mediation analysis represents a crucial methodological framework for addressing these mechanistic questions, as it can decompose total effects into direct and indirect components, thereby elucidating whether metabolites influence cancer risk through inflammatory pathways or via alternative mechanisms [22]. Traditional observational studies cannot adequately address these mechanistic relationships, making mediation analysis essential for understanding the causal pathways linking metabolic dysregulation to oral cancer development through inflammatory mediators [23]. The integration of metabolomics and inflammatory factor analyses within a unified framework to understand oral cancer pathogenesis has not been adequately explored, particularly regarding the potential for inflammatory factors to mediate relationships between metabolic perturbations and cancer development [24]. Furthermore, the application of advanced causal inference methods, particularly MR, to oral cancer research has been limited compared to other cancer types [25]. Mediation analysis techniques, which can elucidate mechanistic pathways between exposures and outcomes, have been underutilized in oral cancer research, representing a significant gap in understanding how metabolites might influence cancer risk through inflammatory pathways [26].

To address these critical knowledge gaps, we conducted a comprehensive MR study combined with mediation analysis to investigate the causal relationships between plasma metabolites, inflammatory factors, and oral cancer risk. Given the limited prior research examining comprehensive metabolomic profiles in oral cancer using causal inference methods, our study employs MR methodology to systematically evaluate the causal effects of 1400 circulating metabolites on oral cancer development while simultaneously exploring the mediating roles of 91 inflammatory factors in these relationships. This comprehensive approach represents one of the extensive applications of metabolomic MR analysis specifically focused on oral cancer. We utilized summary statistics from large‐scale genome‐wide association studies (GWAS), including metabolites from the Canadian Longitudinal Study on Aging (CLSA) cohort (8091 individuals) and oral cancer data from the IEU OpenGWAS database (172,171 participants, including 157 cases and 172,016 controls) [27]. Our analytical framework employed bidirectional MR analyses with rigorous quality control measures, including instrument variable selection with stringent statistical thresholds (*p* < 5 × 10^−6^), linkage disequilibrium (LD) clumping, and multiple MR methods (inverse variance weighted [IVW], MR–Egger, and weighted median) to ensure robust causal estimates [28]. Comprehensive sensitivity analyses were conducted, including heterogeneity testing, horizontal pleiotropy evaluation, and Steiger directionality tests to confirm causal directions [29]. Our mediation analysis framework decomposed total effects into direct and indirect components, quantifying the proportion of metabolite effects on oral cancer risk operating through inflammatory pathways [30]. To validate key MR findings, in vitro experiments were conducted using CAL‐27 cells with aspartate treatment and CCL11 overexpression models. Cellular malignant phenotypes and inflammatory responses were evaluated through functional assays and molecular analyses, and NF‐*κ*B and MAPK signaling pathway activation was assessed to explore upstream regulatory mechanisms. This innovative approach provides novel insights into biological pathways linking metabolic perturbations to oral cancer development and identifies potential therapeutic targets, with important implications for precision medicine approaches and biomarker development [31].

## 2. Materials and Methods

### 2.1. Data Sources and Study Design

We conducted a comprehensive two‐sample MR analysis combined with mediation analysis to investigate the causal relationships between metabolites, inflammatory factors, and oral cancer risk. Genetic data for metabolites were obtained from the CLSA cohort, comprising 8091 individuals with measurements of 1091 metabolites and 109 metabolite ratios, totaling 1400 metabolite GWAS datasets (GCST90200159‐GCST90200814). Summary statistics for oral cavity cancer were retrieved from the IEU OpenGWAS database (ID: ieu‐b‐4961), including 172,171 participants (157 oral cancer cases and 172,016 controls) of European ancestry with 7,721,107 single nucleotide polymorphisms (SNPs). Inflammatory factor data were sourced from 91 plasma protein quantitative trait loci (pQTL) studies measured using the Olink Target platform (GCST90274758‐GCST90274848). All GWAS summary statistics were downloaded from the GWAS Catalog database (https://www.ebi.ac.uk/gwas/). The sample sizes utilized in this study provide adequate statistical power for detecting clinically relevant effect sizes in MR analyses. With 8091 participants for metabolite exposures and 172,171 participants for oral cancer outcomes, our study achieves sufficient power (> 80%) to detect odds ratios (ORs) of 1.10 or greater per standard deviation (SD) change in metabolite levels, assuming genome‐wide significance thresholds and typical instrumental variable strengths observed in metabolomic GWAS. The study design followed a bidirectional approach to distinguish whether metabolite changes caused oral cancer or vice versa. Ethical approval was not required as this study utilized publicly available summary‐level data from previously published GWAS.

### 2.2. Instrument Variable Selection

Genetic instruments for each exposure were selected using the extract_instruments function from the TwoSampleMR R package with a genome‐wide significance threshold (*p* < 5 × 10^−8^). To ensure instrument strength, we calculated *F*‐statistics for each genetic variant, with *F* < 10 indicating weak instruments that were subsequently excluded from the analysis. The *F*‐statistic threshold of 10 is widely accepted in MR studies as it ensures genetic variants explain at least 1% of exposure variance, effectively minimizing weak instrument bias that could lead to unreliable causal estimates. SNPs associated with the outcome variables were extracted using the extract_outcome_data function, ensuring harmonization between exposure and outcome datasets. During data harmonization, missing instrumental SNPs were handled using a standardized protocol, where proxy SNPs with LD *r* ^2^ > 0.8 were identified using the European reference panel when original variants were unavailable in the outcome dataset. SNPs without suitable proxies were excluded from the analysis, and exposures retaining fewer than three instrumental variables after proxy substitution and missing data exclusion were removed from subsequent analyses to maintain adequate statistical power. All data harmonization procedures were performed using the harmonise_data function, which automatically resolved allele alignment, strand orientation, and effect direction consistency across datasets. LD clumping was performed with an *r* ^2^ threshold of 0.001 and a distance window of 10,000 kb to obtain independent genetic variants. Palindromic SNPs with intermediate allele frequencies were removed to avoid strand ambiguity issues. The Steiger directionality test was applied to confirm the correct causal direction and exclude SNPs that showed stronger associations with the outcome than the exposure. Exposures with fewer than three independent instruments were excluded from subsequent analyses to ensure adequate statistical power. All instrument variables were required to satisfy the three core assumptions of MR: relevance, independence, and exclusion restriction.

### 2.3. MR Analysis

Bidirectional two‐sample MR analyses were conducted to examine causal relationships between 1400 metabolites and oral cancer using the TwoSampleMR package in R. Effect estimates were harmonized using the harmonise_data function to ensure consistent effect directions and allele coding across exposure and outcome datasets. Five complementary MR methods were employed to estimate causal effects: IVW, MR–Egger regression, weighted median, simple mode, and weighted mode methods. The IVW method served as the primary analysis, providing the most precise estimates under the assumption of no horizontal pleiotropy. MR–Egger regression was used to detect and adjust for directional pleiotropy, while the weighted median method provided robust estimates when up to 50% of instruments were invalid. Mode‐based methods offered additional robustness by identifying the most common causal estimate across genetic variants. Results were expressed as ORs with 95% confidence intervals, where OR > 1 indicated increased risk and OR < 1 indicated decreased risk. Statistical significance was defined as *p* < 0.05 for the IVW method, which was considered the primary evidence for causal associations.

### 2.4. Sensitivity Analyses

Comprehensive sensitivity analyses were performed to assess the validity and robustness of MR findings using multiple statistical approaches. Heterogeneity among genetic instruments was evaluated using Cochran′s *Q* test implemented through the mr_heterogeneity function, with significant heterogeneity (*p* < 0.05) suggesting potential violations of MR assumptions. Horizontal pleiotropy was assessed using the MR–Egger intercept test via the mr_pleiotropy_test function, where a nonzero intercept (*p* < 0.05) indicated the presence of directional pleiotropy. Leave‐one‐out analyses were conducted using the mr_leaveoneout function to identify influential SNPs that might drive the observed associations. This analysis sequentially removed each genetic variant and recalculated the effect estimate to assess result stability. Steiger filtering was applied to ensure that genetic variants had stronger associations with the exposure than the outcome, thereby confirming the assumed causal direction. Funnel plots were generated to visualize potential asymmetry and assess the randomness of genetic instrument effects. Results were considered robust when consistent across different MR methods and when sensitivity tests showed no evidence of pleiotropy or heterogeneity.

### 2.5. Assessment of Mediation Effect Direction

**Figure 1:**
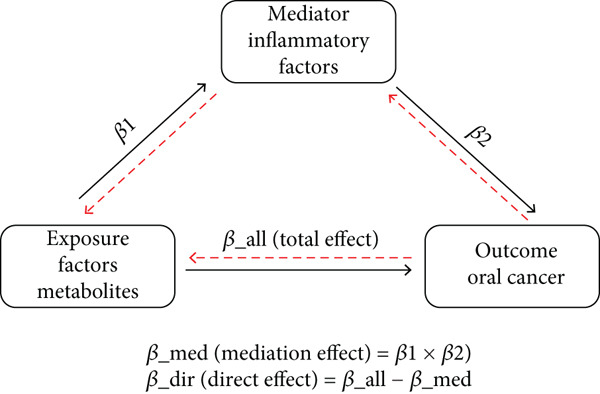
Mediation Mendelian randomization framework. Conceptual model showing causal pathways between metabolites (exposure), inflammatory factors (mediator), and oral cancer (outcome). *β*1: effect of metabolites on inflammatory factors; *β*2: effect of inflammatory factors on oral cancer; *β*_*a* *l* *l*: total effect of metabolites on oral cancer. *M* *e* *d* *i* *a* *t* *i* *o* *n* *e* *f* *f* *e* *c* *t* = *β*1 × *β*2; *d* *i* *r* *e* *c* *t* *e* *f* *f* *e* *c* *t* = *β*_*a* *l* *l* − *β*_*m* *e* *d*.

MR analysis was conducted to evaluate the mediating role of 91 inflammatory factors in the causal pathway between metabolites and oral cancer. The analytical framework followed a three‐node mediation model as illustrated in Figure 1, where metabolites served as exposure factors, inflammatory factors functioned as mediators, and oral cancer represented the outcome variable. As depicted in Figure 1, the mediation model encompasses three distinct pathways: the direct pathway from exposure to outcome (*β*_all, representing total effect), the indirect pathway through the mediator (*β*1 × *β*2, constituting the mediation effect), and the residual direct effect (*β*_direct) after accounting for mediation. Six separate MR assessments were systematically performed to establish valid causal relationships and ensure proper directionality: (1) metabolites → oral cancer, (2) oral cancer → metabolites (with Steiger filtering applied to exclude reverse causation), (3) inflammatory factors → oral cancer, (4) oral cancer → inflammatory factors (with Steiger filtering applied), (5) metabolites → inflammatory factors, and (6) inflammatory factors → metabolites (with Steiger filtering applied). Following the theoretical framework presented in Figure 1, the mediation effect was calculated using *β*_mediation = *β*1 × *β*2, where *β*1 represents the effect of metabolites on inflammatory factors and *β*2 represents the effect of inflammatory factors on oral cancer. The direct effect was calculated as *β*_direct = *β*_all − *β*_mediation, and the proportion of mediation was determined as *β*_mediation_ratio = *β*_mediation/*β*_all, where *β*_all represents the total effect of metabolites on oral cancer as shown by the horizontal pathway in Figure 1.

### 2.6. Cell Culture

CAL‐27 human oral squamous cell carcinoma cells were obtained from China Infrastructure of Cell Line Resource and cultured in Dulbecco′s Modified Eagle Medium (DMEM, Sigma‐Aldrich, United States) containing 10% fetal bovine serum (FBS, Servicebio, China) and 1% antibiotic solution (100 U/mL penicillin and 100 *μ*g/mL streptomycin, Sigma‐Aldrich, United States). Cells were maintained at 37°C in a humidified incubator with 5% CO_2_ atmosphere. Subculturing was performed using 0.25% trypsin‐EDTA (Sigma‐Aldrich, United States) when cells reached 80%–90% confluence. Culture medium was replaced every 2–3 days, and cell morphology and growth were monitored throughout the cultivation period.

### 2.7. Lentiviral Transduction

A human CCL11 cDNA ORF clone (*Homo sapiens*; GenScript, United States) was used in this study and subcloned into the mammalian expression vector pLenti‐GIII‐CMV‐Luc‐2A‐Puro (Applied Biological Materials, ABM, Canada). The resulting construct was cotransfected with the packaging plasmids psPAX2 and pMD2.G into HEK293T cells to produce lentiviral particles. Viral supernatants were collected and filtered for subsequent infection. CAL‐27 cells were then infected with the CCL11‐expressing lentivirus to generate the CCL11‐overexpressing cell line (oe‐CCL11), whereas cells infected with the empty pLenti‐GIII‐CMV‐Luc‐2A‐Puro vector served as the negative control (oe‐NC). Forty‐eight hours after infection, puromycin (2 *μ*g/mL) was applied for 5–7 days to select stable cell populations with integrated constructs.

### 2.8. Aspartate Treatment

L‐Aspartate (Asp, Sigma‐Aldrich, United States) was dissolved in sterile PBS, adjusted to pH 7.2–7.4 with NaOH, and prepared as a 100 mM stock solution, which was sterile‐filtered (0.22 *μ*m) and diluted in complete medium to final concentrations of 0, 0.5, 1, and 5 mM. CAL‐27 cells were allowed to adhere for 24 h and then exposed to the indicated Asp concentrations. Cells were collected after 6, 12, and 48 h to assess CCL11 mRNA expression, and culture supernatants were harvested at 24 and 48 h to quantify extracellular CCL11 protein by ELISA. Based on these concentration–response experiments, 5 mM Asp was selected for subsequent functional assays. For these experiments, cells were assigned to four groups (oe‐NC, oe‐CCL11, oe‐CCL11 + Asp, and Asp), treated with 5 mM Asp for 24 h, and then used for subsequent experiments to evaluate the effects of Asp on CCL11‐mediated cellular phenotypes.

### 2.9. Real‐Time PCR Analysis

Total RNA extraction from CAL‐27 cells was performed using TRIzol reagent, followed by cDNA synthesis with EasyScript First‐Strand SuperMix (TransGen Biotech, China). Quantitative PCR was conducted using SYBR Green SuperMix (Yeasen, China) on an ABI PRISM 7500 system (Applied Biosystems, United States) with specific primers for CCL11 (forward, 5 ^′^‐AAAGCTCACACCTTCAGCCT‐3 ^′^ and reverse, 5 ^′^‐TTTCTGGGGACATTTGCCAC‐3 ^′^) and GAPDH (forward, 5 ^′^‐GCAAATTCCATGGCACCG‐3 ^′^ and reverse, 5 ^′^‐TCGCCCCACTGATTTTGG‐3 ^′^) as reference genes. Thermal cycling included 50°C for 2 min and 95°C for 2 min, followed by 40 cycles of 95°C (15 s) and 60°C (32 s). Relative expression levels were calculated using the 2^−*Δ* *Δ* *C*^ method with GAPDH normalization, and all experiments were performed in triplicate.

### 2.10. ELISA

Culture supernatants were collected at 24 and 48 h after treatment, centrifuged at 1000 × g for 10 min to remove cellular debris, and stored at −80°C until analysis. Concentrations of CCL11, IL‐6, IL‐1*β*, and TNF‐*α* in the supernatants were quantified using human ELISA kits (Sigma‐Aldrich, United States) according to the manufacturer′s instructions. Absorbance was measured using a microplate reader (Thermo Fisher Scientific, United States), and concentrations of CCL11, IL‐6, IL‐1*β*, and TNF‐*α* were calculated from a standard curve.

### 2.11. Metabolic Viability Assessment

CAL‐27 cells from oe‐NC, oe‐CCL11, oe‐CCL11 + Asp, and Asp groups were plated at 5000–10,000 cells/well in 96‐well plates. Following 24‐h incubation at 37°C with 5% CO_2_, CCK8 reagent (Dojindo, Japan) was added at 10% concentration for 1–4 h. Absorbance at 450 nm was measured using a microplate reader (Thermo Fisher Scientific, United States), with viability normalized to untreated controls (100%). Experiments were performed in triplicate.

### 2.12. Clonogenic Survival Analysis

CAL‐27 cells (oe‐NC, oe‐CCL11, oe‐CCL11 + Asp, and Asp groups) were seeded at 200–500 cells/well in six‐well plates and cultured for 2–3 weeks at 37°C in 5% CO_2_ with medium changes every 2–3 days. Colonies were fixed with 4% formaldehyde, stained with 0.1% crystal violet, and counted. Plating efficiency (PE) and survival fraction (SF) were calculated using the following formulas: PE = (number of colonies in control group/number of cells seeded) × 100*%* and SF = (number of colonies in treated group/(number of cells seeded × PE)) × 100*%*. All assays included triplicate measurements.

### 2.13. Wound Healing Assay

Cell migration ability was evaluated through a wound healing assay using CAL‐27 cells in four groups: oe‐NC, oe‐CCL11, oe‐CCL11 + Asp, and Asp treatment. Confluent monolayers in six‐well plates were scratched using sterile 200 *μ*L tips, washed with PBS, and replenished with fresh medium. Gap closure was documented at 0 and 24 h via inverted microscopy (Olympus, Japan) and quantified using ImageJ software. Migration rate was calculated as [(initial width − 24 h width)/initial width] × 100*%*, normalized to controls. All experiments were performed in triplicate.

### 2.14. Transwell Matrigel Invasion Assay

CAL‐27 populations (oe‐NC, oe‐CCL11, oe‐CCL11 + Asp, and Asp treatments) were evaluated using Matrigel‐coated Transwell inserts with 8.0‐*μ*m pores (BD Biosciences, United States). Cells (1 × 10^5^) in serum‐free medium were added to the upper chambers, with complete medium in the lower chambers as a chemoattractant. After 24‐h incubation, invaded cells were fixed with 4% paraformaldehyde, stained with 0.1% crystal violet, and quantified in five random fields at 200× magnification using ImageJ software.

### 2.15. Western Blot Analysis

Total proteins were extracted from CAL‐27 groups (oe‐NC, oe‐CCL11, oe‐CCL11 + Asp, and Asp) using RIPA buffer and quantified by BCA assay (Thermo Fisher Scientific, United States). Proteins were separated on 12% SDS‐PAGE gels and transferred to PVDF membranes (Millipore, United States). After blocking with 5% milk, membranes were incubated with primary antibodies (#ab32536, #ab76302, #ab170099, #ab195049, #ab184699, #ab201015, Abcam, United Kingdom) overnight at 4°C, followed by HRP‐conjugated secondary antibodies (Jackson ImmunoResearch, United States). Proteins were detected using enhanced chemiluminescence (Bio‐Rad, United States) and quantified with ImageJ, normalized to GAPDH controls.

### 2.16. Statistical Analysis

Statistical analyses were performed using R Version 4.4.1 with the following packages: TwoSampleMR (Version 0.6.14) for MR analyses, ggplot2 (Version 1.5.2) for data visualization, and forestplot (Version 1.1.6) for forest plot generation. Data processing and harmonization were conducted using base R functions and tidyverse packages. All visualizations, including scatter plots, forest plots, funnel plots, and leave‐one‐out plots, were generated using standardized functions within the TwoSampleMR framework. Statistical significance was set at *p* < 0.05 for all analyses, and multiple testing correction was applied where appropriate using the Benjamini–Hochberg false discovery rate method. Effect estimates are presented with 95% confidence intervals throughout the manuscript. Reproducible analysis scripts are available upon reasonable request to ensure transparency and replicability of findings.

For in vitro experiments, data are expressed as mean ± SD, derived from a minimum of three independent experiments. Statistical comparisons between two conditions were performed using an unpaired two‐tailed Student′s *t* test. For analyses involving more than two groups, one‐way ANOVA followed by Tukey′s multiple‐comparison procedure was applied. A *p* value below 0.05 was interpreted as statistically significant.

## 3. Results

### 3.1. Causal Relationships Between Metabolites and Oral Cancer

**Figure 2:**
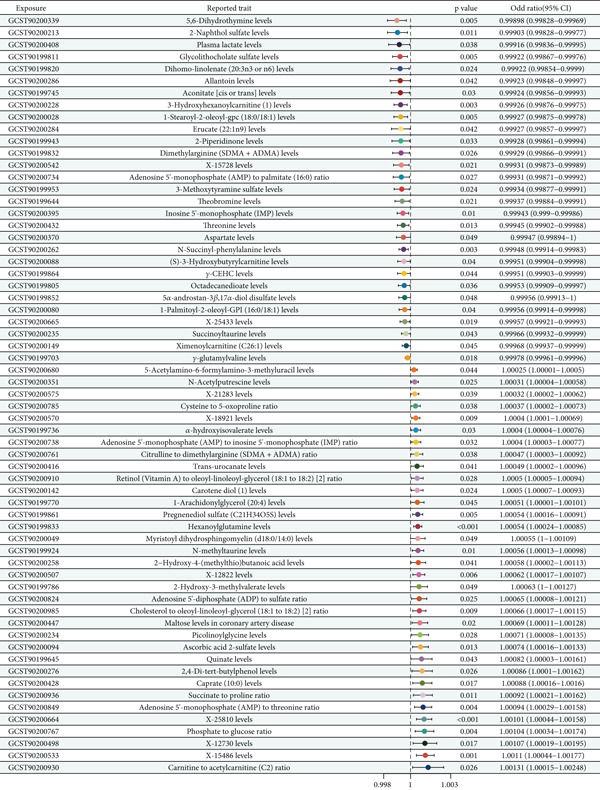
Forest plot of causal relationships between metabolites and oral cancer. The forest plot displays odds ratios (ORs) and 95% confidence intervals (CIs) for 61 metabolites showing significant causal relationships with oral cancer (*p* < 5 × 10^−6^). Metabolites positioned to the left of the vertical reference line (OR < 1) represent protective factors (*n* = 29, 47.5%), while those to the right (OR > 1) represent risk factors (*n* = 32, 52.5%). Each horizontal line represents the 95% CI for individual metabolites, with the point estimate indicated by the center marker. GCST codes identify specific metabolites from the GWAS catalog. *p* values are displayed for each association.

Under screening criteria of *p* < 5 × 10^−6^, clump = TRUE, *r* ^2^ = 0.001, kb = 10,000, *F* > 10, and SNP ≥ 1, MR analysis identified 61 metabolites with significant causal relationships with oral cancer, presented in Figure 2′s forest plot. Among these 61 metabolites, 29 (47.5%) demonstrated protective effects (OR < 1, left of the vertical reference line in Figure 2), with the strongest being 5,6‐dihydrothymine (OR = 0.99898, 95% CI: 0.99828–0.99969, *p* = 0.005), followed by 2‐naphthol sulfate (OR = 0.99903, *p* = 0.011) and plasma lactate (OR = 0.99916, *p* = 0.038). These protective metabolites in Figure 2 include theobromine, gamma‐glutamylvaline, aconitate, octadecanedioate, and others, suggesting they may reduce oral cancer risk through metabolic regulation and immune function enhancement. Conversely, 32 metabolites (52.5%) exhibited risk‐enhancing effects (OR > 1, right of the vertical reference line in Figure 2), with the strongest being carnitine‐to‐acetylcarnitine ratio (OR = 1.00131, *p* = 0.026), followed by phosphate‐to‐glucose ratio (OR = 1.0011, *p* = 0.001) and AMP‐to‐threonine ratio (OR = 1.00107, *p* = 0.017). These risk‐enhancing metabolites in Figure 2 include quinate, alpha‐hydroxyisovalerate, 1‐arachidonylglycerol, and various metabolite ratios, indicating that metabolic abnormalities increase oral cancer risk. Figure 2′s forest plot clearly demonstrates the effect direction, size, and statistical significance of these metabolites.

### 3.2. Exposure–Outcome Correlation Analysis Results

**Figure 3:**
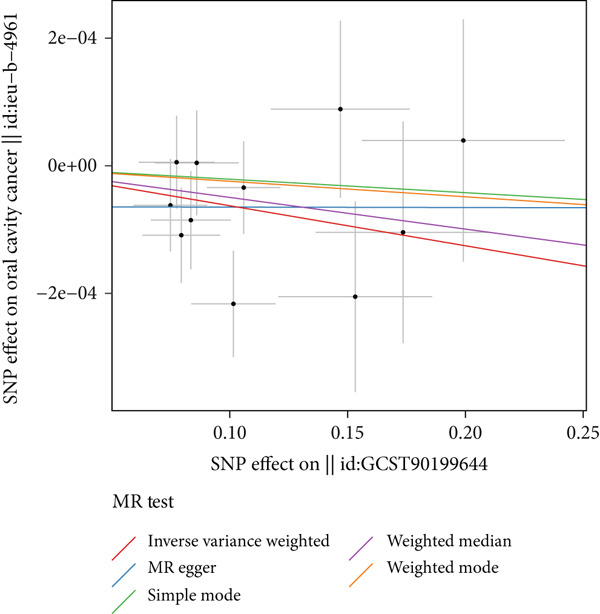
Scatter plot of SNP effects for metabolite–oral cancer causal relationships. SNP effects on metabolites (*x*‐axis) versus oral cancer outcomes (*y*‐axis), with colored lines representing different MR algorithms (IVW, MR–Egger, simple mode, weighted median, and weighted mode). Positive slopes indicate risk factors (OR > 1), and negative slopes indicate protective factors (OR < 1). Nonzero intercepts may suggest horizontal pleiotropy.

To further validate the strength and direction of causal relationships between metabolites and oral cancer, correlation scatter plots based on SNP effects were constructed for visualization analysis. As shown in Figure 3, the scatter plot presents SNP effects on exposure factors along the *x*‐axis and SNP effects on oral cancer outcomes along the *y*‐axis, with each scatter point representing an independent genetic instrumental variable. Different colored lines display the fitting results of various MR algorithms, including IVW, MR–Egger, simple mode, weighted median, and weighted mode methods. Clear observation of Figure 3 reveals that individual SNP data points exhibit regular distribution patterns around different fitting lines, with the various algorithmic fitting lines demonstrating good consistency in Figure 3, thereby enhancing the reliability of causal inference. The slopes of fitting lines in Figure 3 intuitively reflect the direction of causal relationships: positive slopes indicate that corresponding metabolites serve as risk factors for oral cancer, manifested in Figure 3 as ascending trends from lower‐left to upper‐right; negative slopes indicate that metabolites possess protective effects, presenting descending trends from upper‐left to lower‐right in Figure 3. Some fitting lines in Figure 3 exhibit nonzero intercepts, particularly evident in MR–Egger regression lines, suggesting the potential presence of other confounding factors or horizontal pleiotropy. Through systematic analysis of Figure 3 scatter plots, we further confirmed that 29 metabolites as protective factors (OR < 1) demonstrated significant negative causal relationships with oral cancer, and 32 metabolites as risk factors (OR > 1) showed significant positive causal relationships with oral cancer. The distribution patterns of SNP effects in Figure 3 and the consistency of multialgorithmic fitting lines provided robust visualization validation for the forest plot results, while the linear relationship strength demonstrated in Figure 3 reflects the robustness of causal associations between various metabolites and oral cancer.

### 3.3. Instrumental Variable–Outcome Effect Estimation

**Figure 4:**
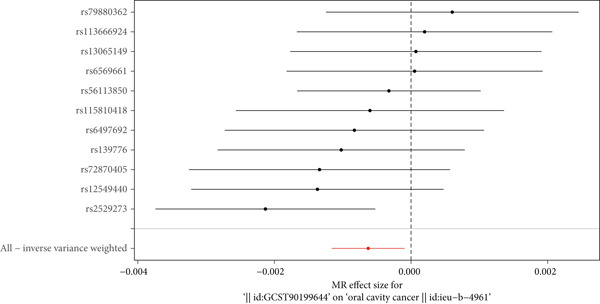
Forest plot of individual SNP effects on metabolite–oral cancer causal relationships. Each horizontal line represents the effect estimate and 95% confidence interval for a single SNP using the Wald ratio method. SNP effects to the left of the vertical reference line indicate protective effects, while effects to the right indicate risk‐enhancing effects. Lines crossing the reference line indicate nonsignificant results. The bottom entry shows the overall IVW estimate integrating all individual SNP effects. rs numbers identify specific genetic variants used as instrumental variables.

To comprehensively evaluate the diagnostic efficacy of individual SNP loci in predicting exposure factor effects on oral cancer outcomes, individual SNP forest plots were constructed to demonstrate the impact of each SNP locus on oral cancer risk. As illustrated in Figure 4, the forest plot displays individual SNP effects calculated using the Wald ratio method, with each horizontal line representing the effect estimate and confidence interval for a single genetic instrument. Figure 4 clearly delineates SNP loci into protective factors (OR < 1) and risk factors (OR > 1) based on their position relative to the vertical reference line. Through systematic examination of forest plots for each exposure factor, Figure 4 demonstrates that 29 metabolites function as protective factors while 32 metabolites serve as risk factors with significant causal relationships to oral cancer. The visual representation in Figure 4 allows immediate identification of effect directionality: lines positioned entirely left of zero indicate increased exposure reduces outcome risk, lines entirely right indicate increased exposure elevates risk, while lines crossing the reference line suggest nonsignificant results. Furthermore, Figure 4 presents the overall IVW model effect at the bottom, comprehensively considering all SNP loci′s influence and providing a global perspective for evaluating the relationship between genetic instruments and oral cancer. The systematic arrangement of individual SNP effects in Figure 4 offers detailed insight into genetic instrument heterogeneity and their collective contribution to causal inference, enabling a comprehensive understanding of complex relationships between different metabolites and oral cancer risk.

### 3.4. MR Randomness Assessment Results

**Figure 5:**
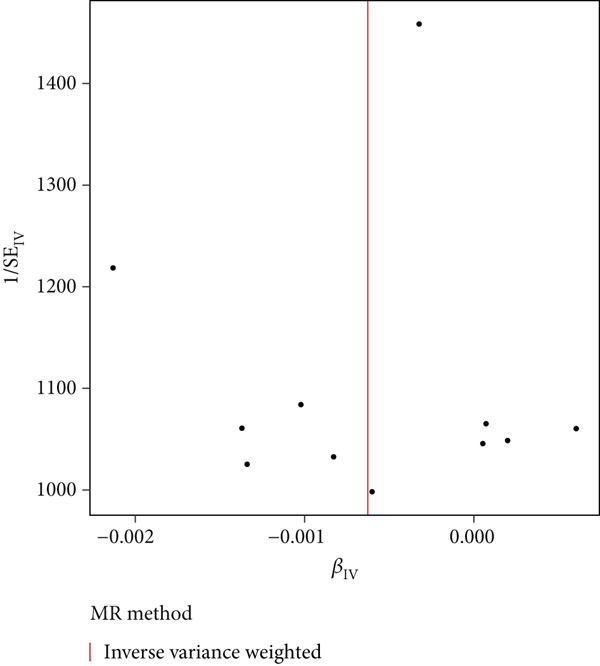
Funnel plot for assessment of MR study randomness and potential bias. Effect estimates (*β* coefficients) on the *x*‐axis and inverse SEs (1/SE) on the *y*‐axis, with each point representing an individual SNP. Vertical line indicates the overall IVW effect estimate. Symmetric distribution suggests the absence of systematic bias and adherence to random assortment principles. Higher points represent more precise estimates. The symmetric pattern supports the MR assumption validity.

To evaluate analysis randomness and confirm the MR study conformity to random assortment principles, funnel plots were employed as statistical visualization tools. Funnel plots combined the effect estimates (*β* coefficients) of individual SNPs with their standard errors (SEs). As demonstrated in Figure 5, SNP distribution exhibited approximately symmetric patterns on both sides of the central reference line following IVW processing. This symmetric distribution in Figure 5 strongly indicates that instrumental variables were not subject to significant systematic bias but conformed to expected random distribution patterns. The visual symmetry in Figure 5 provides evidence that genetic instruments are appropriately distributed around the overall effect estimate, with higher SE studies (positioned higher in Figure 5) showing natural variation while lower SE studies (positioned lower in Figure 5) cluster more closely around the central estimate. Sensitivity analyses identified potential outliers or bias sources, with consistent patterns supporting the validity of the symmetric distribution shown in Figure 5 and reinforcing confidence in the absence of publication bias. In conclusion, the visualization provided by Figure 5, combined with sensitivity analyses, demonstrates that the MR study exhibits good randomness characteristics and conforms to random assortment principles, as evidenced by the balanced distribution of genetic instruments in Figure 5, providing a solid statistical foundation for exploring causal relationships between metabolites and oral cancer.

### 3.5. MR Heterogeneity Analysis Results

Comprehensive heterogeneity analysis was performed to assess the consistency of causal effect estimates across genetic instruments for all 61 metabolite–oral cancer associations. As presented in Table 1, *Q*_*p*val values from Cochran′s *Q* test serve as critical indicators for determining significant heterogeneity between instrumental variables, with values > 0.05 indicating the absence of significant heterogeneity. Table 1 demonstrates that all *Q*_*p*val values exceeded the 0.05 threshold, ranging from 0.0602 to 0.9725, with 59 out of 61 associations (96.7%) showing *Q*_*p*val values > 0.1. The consistent pattern observed in Table 1 provides robust evidence that no significant heterogeneity exists among genetic instruments, despite employing a two‐sample MR design with data from different population cohorts. This finding, as demonstrated across all entries in Table 1, indicates that potential confounding factors such as population stratification or methodological differences did not substantially influence the analytical outcomes, thereby enhancing confidence in the identified causal relationships between metabolites and oral cancer and validating the reliability of the two‐sample MR approach.

**Table 1:** Results of MR heterogeneity analysis for metabolite–oral cancer associations. *Q*_*p*val represents the *p* value from Cochran′s *Q* test for heterogeneity using the IVW method, where values > 0.05 indicate the absence of significant heterogeneity. All 61 metabolite associations demonstrated *Q*_*p*val > 0.05, confirming homogeneity of effect estimates and validating two‐sample MR design reliability. GCST codes identify metabolite exposures; ieu‐b‐4961 represents the oral cancer outcome dataset.

**id.exposure**	**id.outcome**	**Method**	**Q**_**p** **v** **a** **l**
GCST90199644	ieu‐b‐4961	Inverse variance weighted	0.585258918
GCST90199645	ieu‐b‐4961	Inverse variance weighted	0.881251407
GCST90199701	ieu‐b‐4961	Inverse variance weighted	0.782204259
GCST90199716	ieu‐b‐4961	Inverse variance weighted	0.651261894
GCST90199745	ieu‐b‐4961	Inverse variance weighted	0.718152447
GCST90199770	ieu‐b‐4961	Inverse variance weighted	0.229715049
GCST90199786	ieu‐b‐4961	Inverse variance weighted	0.16289662
GCST90199805	ieu‐b‐4961	Inverse variance weighted	0.917879159
GCST90199811	ieu‐b‐4961	Inverse variance weighted	0.516588722
GCST90199820	ieu‐b‐4961	Inverse variance weighted	0.847240802
GCST90199812	ieu‐b‐4961	Inverse variance weighted	0.566181418
GCST90199811	ieu‐b‐4961	Inverse variance weighted	0.806679286
GCST90199852	ieu‐b‐4961	Inverse variance weighted	0.796809792
GCST90199861	ieu‐b‐4961	Inverse variance weighted	0.626661466
GCST90199864	ieu‐b‐4961	Inverse variance weighted	0.772848272
GCST90199924	ieu‐b‐4961	Inverse variance weighted	0.927621954
GCST90199941	ieu‐b‐4961	Inverse variance weighted	0.454565227
GCST90199951	ieu‐b‐4961	Inverse variance weighted	0.792209511
GCST90200028	ieu‐b‐4961	Inverse variance weighted	0.418794107
GCST90200049	ieu‐b‐4961	Inverse variance weighted	0.898125426
GCST90200080	ieu‐b‐4961	Inverse variance weighted	0.811192919
GCST90200088	ieu‐b‐4961	Inverse variance weighted	0.487912459
GCST90200094	ieu‐b‐4961	Inverse variance weighted	0.961486458
GCST90200142	ieu‐b‐4961	Inverse variance weighted	0.744164021
GCST90200149	ieu‐b‐4961	Inverse variance weighted	0.444972276
GCST90200211	ieu‐b‐4961	Inverse variance weighted	0.828506621
GCST90200228	ieu‐b‐4961	Inverse variance weighted	0.718798151
GCST90200214	ieu‐b‐4961	Inverse variance weighted	0.817818058
GCST90200215	ieu‐b‐4961	Inverse variance weighted	0.526471074
GCST90200258	ieu‐b‐4961	Inverse variance weighted	0.115710847
GCST90200262	ieu‐b‐4961	Inverse variance weighted	0.414111204
GCST90200276	ieu‐b‐4961	Inverse variance weighted	0.615188011
GCST90200284	ieu‐b‐4961	Inverse variance weighted	0.588001912
GCST90200286	ieu‐b‐4961	Inverse variance weighted	0.755409611
GCST90200119	ieu‐b‐4961	Inverse variance weighted	0.541851279
GCST90200151	ieu‐b‐4961	Inverse variance weighted	0.758801019
GCST90200170	ieu‐b‐4961	Inverse variance weighted	0.888848618
GCST90200195	ieu‐b‐4961	Inverse variance weighted	0.751871008
GCST90200408	ieu‐b‐4961	Inverse variance weighted	0.972514192
GCST90200416	ieu‐b‐4961	Inverse variance weighted	0.254761266
GCST90200428	ieu‐b‐4961	Inverse variance weighted	0.410456514
GCST90200412	ieu‐b‐4961	Inverse variance weighted	0.295664627
GCST90200447	ieu‐b‐4961	Inverse variance weighted	0.701948876
GCST90200498	ieu‐b‐4961	Inverse variance weighted	0.604886519
GCST90200507	ieu‐b‐4961	Inverse variance weighted	0.617608407
GCST90200511	ieu‐b‐4961	Inverse variance weighted	0.549099111
GCST90200542	ieu‐b‐4961	Inverse variance weighted	0.89141885
GCST90200570	ieu‐b‐4961	Inverse variance weighted	0.968101699
GCST90200575	ieu‐b‐4961	Inverse variance weighted	0.815911728
GCST90200664	ieu‐b‐4961	Inverse variance weighted	0.967711911
GCST90200665	ieu‐b‐4961	Inverse variance weighted	0.615822728
GCST90200680	ieu‐b‐4961	Inverse variance weighted	0.792805051
GCST90200714	ieu‐b‐4961	Inverse variance weighted	0.607416807
GCST90200718	ieu‐b‐4961	Inverse variance weighted	0.478259685
GCST90200761	ieu‐b‐4961	Inverse variance weighted	0.669144415
GCST90200767	ieu‐b‐4961	Inverse variance weighted	0.259090571
GCST90200785	ieu‐b‐4961	Inverse variance weighted	0.565616288
GCST90200824	ieu‐b‐4961	Inverse variance weighted	0.418447852
GCST90200849	ieu‐b‐4961	Inverse variance weighted	0.9541885
GCST90200910	ieu‐b‐4961	Inverse variance weighted	0.111125107
GCST90200910	ieu‐b‐4961	Inverse variance weighted	0.450120412
GCST90200916	ieu‐b‐4961	Inverse variance weighted	0.598119099
GCST90200985	ieu‐b‐4961	Inverse variance weighted	0.0602455421

### 3.6. MR Horizontal Pleiotropy Analysis Results

To evaluate potential confounding effects from horizontal pleiotropy, MR–Egger regression analysis was conducted for all 61 metabolite–oral cancer associations using the TwoSampleMR R package. Horizontal pleiotropy occurs when genetic variants influence outcomes through pathways independent of the exposure, potentially compromising causal inference validity. As presented in Table 2, the *p* values from MR–Egger pleiotropy tests serve as critical indicators for detecting pleiotropic effects, with values > 0.05 indicating the absence of significant horizontal pleiotropy. Table 2 demonstrates that all *p* values exceeded the 0.05 significance threshold, ranging from 0.080% to 0.996, with 57 out of 61 associations (93.4%) showing *p* values > 0.2. The consistent results shown in Table 2 confirm that no significant horizontal pleiotropic effects were detected, indicating that the identified causal relationships between metabolites and oral cancer were not confounded by alternative biological pathways, thereby supporting the validity of MR assumptions and the reliability of causal inferences.

**Table 2:** Results of MR horizontal pleiotropy analysis for metabolite–oral cancer associations. MR–Egger pleiotropy test results for 61 metabolite exposures, showing egger_intercept, SE (standard error), and *p*val (*p* value for horizontal pleiotropy testing). *p* values > 0.05 indicate the absence of significant horizontal pleiotropy. All associations demonstrated *p*val > 0.05, confirming that genetic instruments do not influence oral cancer through pathways independent of metabolite exposures.

**id.exposure**	**id.outcome**	**egger_intercept**	**SE**	**p** **v** **a** **l**
GCST90199644	ieu‐b‐4961	−6.41e − 05	9.74e − 05	0.527069718
GCST90199645	ieu‐b‐4961	−1.79e − 05	0.000115822	0.794286187
GCST90199701	ieu‐b‐4961	1.19e − 05	1.70e − 05	0.755124217
GCST90199716	ieu‐b‐4961	−1.12e − 05	5.47e − 05	0.582611427
GCST90199745	ieu‐b‐4961	−7.26e − 05	0.00011915	0.620411181
GCST90199770	ieu‐b‐4961	0.000105612	7.16e − 05	0.201157186
GCST90199786	ieu‐b‐4961	−0.00018118	0.000114747	0.148681957
GCST90199805	ieu‐b‐4961	4.11e − 05	8.46e − 05	0.621824919
GCST90199811	ieu‐b‐4961	0.000141721	0.000119978	0.11696109
GCST90199820	ieu‐b‐4961	−1.22e − 05	0.000124722	0.926507189
GCST90199812	ieu‐b‐4961	−6.20e − 05	8.69e − 05	0.514948222
GCST90199811	ieu‐b‐4961	−2.16e − 05	5.42e − 05	0.701928262
GCST90199852	ieu‐b‐4961	−6.06e − 05	7.21e − 05	0.420765751
GCST90199861	ieu‐b‐4961	2.94e − 05	4.69e − 05	0.516872487
GCST90199864	ieu‐b‐4961	6.21e − 05	9.66e − 05	0.529625678
GCST90199924	ieu‐b‐4961	6.81e − 06	0.000149067	0.96418118
GCST90199941	ieu‐b‐4961	0.000146542	0.000128021	0.295944984
GCST90199951	ieu‐b‐4961	−7.68e − 06	0.000177844	0.967246271
GCST90200028	ieu‐b‐4961	−0.000167122	0.000118968	0.187694565
GCST90200049	ieu‐b‐4961	7.60e − 05	0.000121559	0.554741101
GCST90200080	ieu‐b‐4961	−5.54e − 05	8.59e − 05	0.511457687
GCST90200088	ieu‐b‐4961	2.01e − 05	9.12e − 05	0.812194449
GCST90200094	ieu‐b‐4961	1.45e − 05	0.000119449	0.780777217
GCST90200142	ieu‐b‐4961	2.87e − 05	8.14e − 05	0.742155104
GCST90200149	ieu‐b‐4961	1.71e − 05	5.42e − 05	0.75690542
GCST90200211	ieu‐b‐4961	−5.01e − 05	0.000119191	0.716681511
GCST90200228	ieu‐b‐4961	−0.000111875	9.65e − 05	0.276118111
GCST90200214	ieu‐b‐4961	1.80e − 05	0.000154554	0.814109026
GCST90200215	ieu‐b‐4961	1.45e − 05	5.26e − 05	0.529012879
GCST90200258	ieu‐b‐4961	−1.29e − 05	0.000105151	0.761110919
GCST90200262	ieu‐b‐4961	0.000110259	7.97e − 05	0.218855081
GCST90200276	ieu‐b‐4961	−2.84e − 05	0.000111756	0.840046992
GCST90200284	ieu‐b‐4961	−0.000112812	9.52e − 05	0.221826096
GCST90200286	ieu‐b‐4961	0.000101171	0.000202127	0.611481715
GCST90200119	ieu‐b‐4961	−6.91e − 05	9.91e − 05	0.511527401
GCST90200151	ieu‐b‐4961	2.61e − 05	4.19e − 05	0.544080641
GCST90200170	ieu‐b‐4961	−5.06e − 05	9.10e − 05	0.601528008
GCST90200195	ieu‐b‐4961	−2.79e − 05	0.000106117	0.798820989
GCST90200408	ieu‐b‐4961	−2.06e − 05	0.000189211	0.918667285
GCST90200416	ieu‐b‐4961	6.14e − 05	0.000100668	0.56106545
GCST90200428	ieu‐b‐4961	0.000226694	0.000116167	0.108468289
GCST90200412	ieu‐b‐4961	−0.00014407	7.68e − 05	0.080269004
GCST90200447	ieu‐b‐4961	−0.000151192	9.68e − 05	0.168871561
GCST90200498	ieu‐b‐4961	7.44e − 05	0.000129164	0.622570725
GCST90200507	ieu‐b‐4961	−0.000148991	9.21e − 05	0.18108184
GCST90200511	ieu‐b‐4961	−0.000101669	0.000115011	0.512989582
GCST90200542	ieu‐b‐4961	0.000110187	0.000151252	0.414419176
GCST90200570	ieu‐b‐4961	5.77e − 05	5.20e − 05	0.295941701
GCST90200575	ieu‐b‐4961	2.19e − 07	4.71e − 05	0.996088526
GCST90200664	ieu‐b‐4961	6.01e − 05	0.000114692	0.614691741
GCST90200665	ieu‐b‐4961	−7.06e − 06	5.14e − 05	0.891114866
GCST90200680	ieu‐b‐4961	7.50e − 06	4.11e − 05	0.866158216
GCST90200714	ieu‐b‐4961	1.86e − 05	0.000122971	0.761191647
GCST90200718	ieu‐b‐4961	−1.92e − 05	9.41e − 05	0.6850188
GCST90200761	ieu‐b‐4961	1.12e − 05	7.81e − 05	0.888185107
GCST90200767	ieu‐b‐4961	−8.15e − 05	0.000105555	0.451597474
GCST90200785	ieu‐b‐4961	1.00e − 05	4.84e − 05	0.552001127
GCST90200824	ieu‐b‐4961	4.51e − 05	0.000141467	0.761577617
GCST90200849	ieu‐b‐4961	0.000100648	0.00014774	0.517608486
GCST90200910	ieu‐b‐4961	7.76e − 05	7.14e − 05	0.294241581
GCST90200910	ieu‐b‐4961	0.000158872	0.0004015	0.760128101
GCST90200916	ieu‐b‐4961	−6.11e − 05	0.000161407	0.71592228
GCST90200985	ieu‐b‐4961	0.000117126	8.12e − 05	0.111462011

### 3.7. Leave‐One‐Out Sensitivity Analysis Results

**Figure 6:**
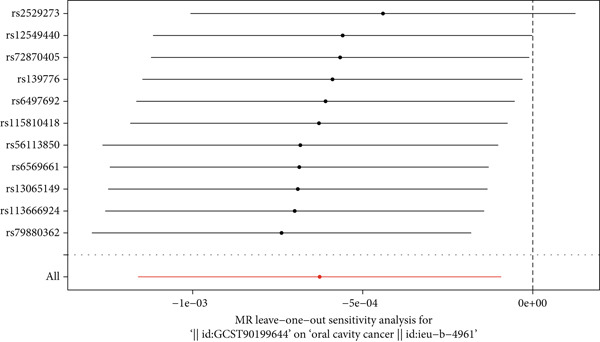
Leave‐one‐out sensitivity analysis forest plot for metabolite–oral cancer associations. Each horizontal line represents the MR effect estimate and 95% confidence interval after excluding one SNP, with rs numbers on the *y*‐axis and effect estimates on the *x*‐axis. The red vertical line indicates the overall pooled effect estimate. Consistent clustering of individual estimates around the overall effect demonstrates that no single SNP disproportionately influences the causal inference, confirming MR analysis robustness.

To comprehensively evaluate the robustness and reliability of the MR analysis results, a leave‐one‐out sensitivity analysis was performed to assess the influence of individual genetic instruments on the overall causal estimates. This systematic approach involved sequentially removing each SNP from the analysis and recalculating the combined effect of the remaining genetic instruments using the IVW method. As illustrated in Figure 6, the leave‐one‐out analysis demonstrated remarkable consistency across all metabolite–oral cancer associations, with no single SNP exerting disproportionate influence on the overall causal estimates. The forest plot presentation in Figure 6 clearly shows that the exclusion of any individual SNP did not result in substantial changes to the effect estimates, as evidenced by the tight clustering of individual SNP effects around the overall pooled estimate (indicated by the red reference line in Figure 6). The visual representation in Figure 6 reveals that the confidence intervals of individual leave‐one‐out estimates largely overlap with the overall effect estimate, confirming the stability of the causal relationships identified. Furthermore, the systematic pattern observed in Figure 6 indicates that no outlying SNPs were driving the observed associations, thereby strengthening confidence in the validity of the MR assumptions. The consistency of results across all leave‐one‐out iterations, as demonstrated in Figure 6, provides compelling evidence that the identified causal relationships between metabolites and oral cancer are robust and not dependent on any single genetic variant. This stability pattern, shown in Figure 6, supports the reliability of the two‐sample MR design and validates the biological plausibility of the metabolite–oral cancer causal pathways identified in this study.

### 3.8. Reverse Causality Assessment Results

To investigate the directionality of causal relationships between metabolites and oral cancer, MR–Steiger analysis was performed using the IVW method. This analysis determines whether metabolites are genuine causal factors for oral cancer development rather than consequences of the disease. As demonstrated in Table 3, all 61 metabolite exposures showed correct causal direction (TRUE) with *p* values < 0.05, indicating statistical significance across all associations. The results in Table 3 provide compelling evidence that metabolites represent genuine causal factors for oral cancer development rather than reverse causation. The highly significant *p* values in Table 3 (ranging from 4.82e − 10 to 4.69e − 285) reinforce the statistical robustness of the directional inference. The comprehensive assessment presented in Table 3 confirms that metabolite variations constitute independent factors for increased oral cancer risk, validating the absence of reverse causation in the identified causal relationships.

**Table 3:** MR–Steiger directionality test results for metabolite–oral cancer associations. Directionality assessment for 61 metabolite exposures showing id.exposure (GCST codes), id.outcome (ieu‐b‐4961), correct_causal_direction (TRUE/FALSE), and steiger_pval (*p* value for directionality testing). All associations demonstrated TRUE directionality with *p* values < 0.05, confirming metabolites are genuine causal factors rather than disease consequences.

**id.exposure**	**id.outcome**	**correct_causal_direction**	**steiger_pval**
GCST90199644	ieu‐b‐4961	TRUE	2.46e − 61
GCST90199645	ieu‐b‐4961	TRUE	1.29e − 28
GCST90199701	ieu‐b‐4961	TRUE	1.15e − 56
GCST90199716	ieu‐b‐4961	TRUE	6.27e − 160
GCST90199745	ieu‐b‐4961	TRUE	1.89e − 41
GCST90199770	ieu‐b‐4961	TRUE	2.17e − 95
GCST90199786	ieu‐b‐4961	TRUE	1.26e − 71
GCST90199805	ieu‐b‐4961	TRUE	1.72e − 97
GCST90199811	ieu‐b‐4961	TRUE	1.49e − 64
GCST90199820	ieu‐b‐4961	TRUE	1.21e − 19
GCST90199812	ieu‐b‐4961	TRUE	6.71e − 49
GCST90199811	ieu‐b‐4961	TRUE	1.66e − 211
GCST90199852	ieu‐b‐4961	TRUE	1.16e − 81
GCST90199861	ieu‐b‐4961	TRUE	4.89e − 179
GCST90199864	ieu‐b‐4961	TRUE	1.01e − 76
GCST90199924	ieu‐b‐4961	TRUE	1.56e − 61
GCST90199941	ieu‐b‐4961	TRUE	1.05e − 18
GCST90199951	ieu‐b‐4961	TRUE	9.07e − 16
GCST90200028	ieu‐b‐4961	TRUE	7.07e − 71
GCST90200049	ieu‐b‐4961	TRUE	1.76e − 65
GCST90200080	ieu‐b‐4961	TRUE	9.99e − 104
GCST90200088	ieu‐b‐4961	TRUE	1.68e − 92
GCST90200094	ieu‐b‐4961	TRUE	5.06e − 51
GCST90200142	ieu‐b‐4961	TRUE	1.50e − 97
GCST90200149	ieu‐b‐4961	TRUE	1.01e − 205
GCST90200211	ieu‐b‐4961	TRUE	1.64e − 29
GCST90200228	ieu‐b‐4961	TRUE	5.25e − 78
GCST90200214	ieu‐b‐4961	TRUE	8.48e − 45
GCST90200215	ieu‐b‐4961	TRUE	2.06e − 165
GCST90200258	ieu‐b‐4961	TRUE	2.41e − 91
GCST90200262	ieu‐b‐4961	TRUE	1.12e − 118
GCST90200276	ieu‐b‐4961	TRUE	4.82e − 10
GCST90200284	ieu‐b‐4961	TRUE	5.77e − 16
GCST90200286	ieu‐b‐4961	TRUE	1.05e − 14
GCST90200119	ieu‐b‐4961	TRUE	6.82e − 16
GCST90200151	ieu‐b‐4961	TRUE	2.05e − 272
GCST90200170	ieu‐b‐4961	TRUE	1.69e − 69
GCST90200195	ieu‐b‐4961	TRUE	2.90e − 52
GCST90200408	ieu‐b‐4961	TRUE	4.41e − 29
GCST90200416	ieu‐b‐4961	TRUE	1.51e − 108
GCST90200428	ieu‐b‐4961	TRUE	2.21e − 14
GCST90200412	ieu‐b‐4961	TRUE	9.10e − 107
GCST90200447	ieu‐b‐4961	TRUE	5.48e − 40
GCST90200498	ieu‐b‐4961	TRUE	5.14e − 20
GCST90200507	ieu‐b‐4961	TRUE	2.48e − 91
GCST90200511	ieu‐b‐4961	TRUE	6.60e − 41
GCST90200542	ieu‐b‐4961	TRUE	1.17e − 50
GCST90200570	ieu‐b‐4961	TRUE	4.77e − 229
GCST90200575	ieu‐b‐4961	TRUE	1.96e − 171
GCST90200664	ieu‐b‐4961	TRUE	1.19e − 51
GCST90200665	ieu‐b‐4961	TRUE	2.02e − 117
GCST90200680	ieu‐b‐4961	TRUE	4.69e − 285
GCST90200714	ieu‐b‐4961	TRUE	4.07e − 47
GCST90200718	ieu‐b‐4961	TRUE	1.47e − 70
GCST90200761	ieu‐b‐4961	TRUE	2.92e − 88
GCST90200767	ieu‐b‐4961	TRUE	8.69e − 46
GCST90200785	ieu‐b‐4961	TRUE	4.65e − 154
GCST90200824	ieu‐b‐4961	TRUE	9.40e − 12
GCST90200849	ieu‐b‐4961	TRUE	1.16e − 42
GCST90200910	ieu‐b‐4961	TRUE	1.12e − 120
GCST90200910	ieu‐b‐4961	TRUE	2.17e − 14
GCST90200916	ieu‐b‐4961	TRUE	8.72e − 17
GCST90200985	ieu‐b‐4961	TRUE	6.11e − 114

### 3.9. Direct Causal Relationships Between Inflammatory Factors and Oral Cancer

**Figure 7:**
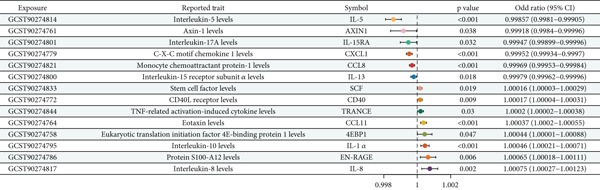
Forest plot of causal relationships between inflammatory factors and oral cancer. Odds ratios (ORs) and 95% confidence intervals for 14 inflammatory factors with significant causal relationships to oral cancer. The vertical reference line at OR = 1.0 distinguishes protective factors (left, OR < 1) from risk factors (right, OR > 1). All associations met the significance threshold *p* < 5 × 10^−6^ and passed sensitivity testing.

Following comprehensive mediation MR analysis using 91 inflammatory factors as potential mediators, 14 inflammatory factors demonstrated significant direct causal relationships with oral cancer under the screening criteria. As illustrated in Figure 7, six inflammatory factors functioned as protective factors (OR < 1), including AXIN1 (OR = 0.99918, *p* = 0.038), CXCL1 (OR = 0.99952, *p* < 0.001), IL‐13 (OR = 0.99979, *p* = 0.018), IL‐15RA (OR = 0.99947, *p* = 0.032), IL‐5 (OR = 0.99857, *p* < 0.001), and CCL8 (OR = 0.99969, *p* < 0.001). These protective factors appear to the left of the vertical reference line in Figure 7, suggesting their role in reducing oral cancer risk through immune regulation. Conversely, eight inflammatory factors exhibited risk‐enhancing effects (OR > 1), including 4EBP1 (OR = 1.00044, *p* = 0.047), CCL11 (OR = 1.00037, *p* < 0.001), CD40 (OR = 1.00017, *p* = 0.009), EN‐RAGE (OR = 1.00065, *p* = 0.006), IL‐1 alpha (OR = 1.00046, *p* < 0.001), IL‐8 (OR = 1.00075, *p* = 0.002), SCF (OR = 1.00016, *p* = 0.019), and TRANCE (OR = 1.0002, *p* = 0.03). These risk factors appear to the right of the reference line in Figure 7, indicating their contribution to increased oral cancer risk. The forest plot in Figure 7 clearly demonstrates the effect direction, magnitude, and statistical significance of all inflammatory factor–oral cancer associations.

### 3.10. Causal Relationships Between Metabolites and Inflammatory Factors

**Figure 8:**
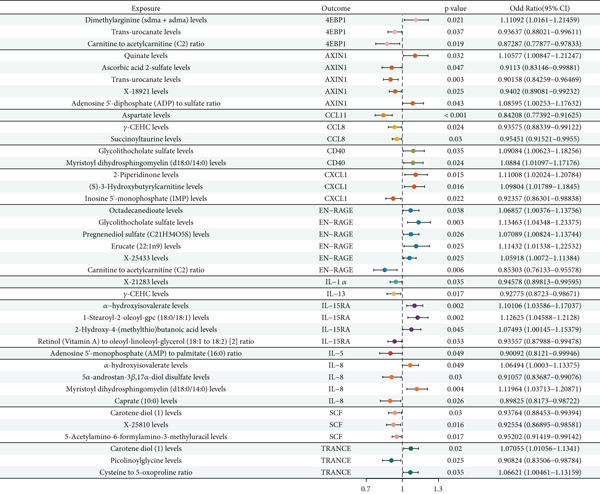
Forest plot of causal relationships between metabolites and inflammatory factors. Odds ratios (ORs) and 95% confidence intervals for metabolite–inflammatory factor associations. Columns show exposure metabolite identifiers, outcome inflammatory factor identifiers, *p* values, and OR values with confidence intervals. The vertical reference line at OR = 1.0 distinguishes protective effects (left) from risk‐enhancing effects (right). All associations met the significance threshold *p* < 5 × 10^−6^.

MR analysis was performed using 61 metabolites as exposure factors and 14 inflammatory factors as outcomes under the screening criteria of *p* < 5 × 10^−6^. The analysis revealed 39 significant metabolite–inflammatory factor associations, as demonstrated in Figure 8. Among the associations shown in Figure 8, several metabolites demonstrated protective effects (OR < 1) on inflammatory factors, including *trans*‐urocanate levels with 4EBP1 (OR = 0.93637, *p* = 0.037), carnitine‐to‐acetylcarnitine ratio with 4EBP1 (OR = 0.87287, *p* = 0.019), and aspartate levels with CCL11 (OR = 0.84208, *p* < 0.001). Conversely, Figure 8 reveals metabolites with risk‐enhancing effects (OR > 1), such as dimethylarginine levels with 4EBP1 (OR = 1.11092, *p* = 0.021) and quinate levels with AXIN1 (OR = 1.10577, *p* = 0.032). The forest plot in Figure 8 demonstrates bidirectional metabolite–inflammatory factor interactions, with effects positioned both left and right of the vertical reference line at OR = 1.0. The visualization in Figure 8 shows that multiple inflammatory factors serve as common targets for various metabolites, suggesting their potential roles as key mediators in metabolite–oral cancer pathways. All associations in Figure 8 were validated through sensitivity analyses and Steiger directionality testing.

### 3.11. Quantification of Mediation Effects in Metabolite–Oral Cancer Associations

To investigate mediating pathways through which metabolites influence oral cancer risk via inflammatory factors, detailed mediation effect calculations were performed. Using the screening criterion of beta_mediation_ratio > 0, 15 significant mediation pathways were identified and presented in Table 4. The results in Table 4 demonstrate that 14 metabolites mediate their effects on oral cancer through eight inflammatory factors, with mediation ratios ranging from 1.4% to 17.4% of the total effect. Clinically, a 1.4%–17.4% mediated proportion indicates that inflammation accounts for a measurable minority of the overall metabolite effect—consistent with the multifactorial etiology of oral cancer—and highlights IL‐8/AXIN1‐centered axes as actionable, additive targets that can be combined with other risk‐modifying strategies. Among the pathways shown in Table 4, *trans*‐urocanate levels demonstrated the highest mediation ratio through AXIN1 (17.4%), followed by 5alpha‐androstan‐3beta,17alpha‐diol disulfate levels through IL‐8 (16.0%), and myristoyl dihydrosphingomyelin levels through IL‐8 (15.5%). Table 4 reveals that IL‐8 and AXIN1 serve as key mediators for multiple metabolites. These modest percentages do not diminish clinical relevance: Even partial mediation can translate into meaningful absolute risk reductions in high‐risk populations, and the nonmediated proportion plausibly reflects parallel noninflammatory routes (e.g., epithelial metabolic reprogramming, oxidative/DNA‐damage processes, and microbiome–host interactions) that warrant future investigation. The mediation pathways identified in Table 4 involve diverse metabolic processes, including lipid metabolism, amino acid metabolism, and vitamin metabolism, suggesting that inflammatory factors serve as common downstream effectors for various metabolic perturbations. The findings in Table 4 provide evidence that metabolites influence oral cancer development through both direct pathways and indirect pathways mediated by inflammatory factors.

**Table 4:** Details of mediation effect calculations for metabolite–oral cancer associations. Mediation analysis results showing exposure metabolites, mediating inflammatory factors, outcome (ieu‐b‐4961), total effect (beta_all), mediation effect (beta_mediation), and mediation ratio (beta_mediation_ratio). Beta_mediation_ratio represents the proportion of the total effect mediated through inflammatory factors. All entries demonstrate positive mediation ratios (> 0), with ratios ranging from 1.4% to 17.4%.

**Exposure**	**Mediation**	**id.outcome**	**beta_all**	**beta_mediation**	**beta_mediation_ratio**
Alpha‐hydroxyisovalerate levels	IL‐8	ieu‐b‐4961	0.000196642	4.71e − 05	0.118774978
5alpha‐androstan‐1beta,17alpha‐diol disulfate levels	IL‐8	ieu‐b‐4961	−0.000419579	−7.01e − 05	0.159581059
Pregnenediol sulfate (C21H14O5S) levels	EN‐RAGE	ieu‐b‐4961	0.000515012	4.41e − 05	0.082726129
2‐Piperidinone levels	CXCL1	ieu‐b‐4961	−0.000724944	−5.01e − 05	0.069126711
1‐Stearoyl‐2‐oleoyl‐gpc (18:0/18:1) levels	IL‐15RA	ieu‐b‐4961	−0.000711512	−6.10e − 05	0.085902944
Myristoyl dihydrosphingomyelin (d18:0/14:0) levels	CD40	ieu‐b‐4961	0.000544956	1.48e − 05	0.027124781
Myristoyl dihydrosphingomyelin (d18:0/14:0) levels	IL‐8	ieu‐b‐4961	0.000544956	8.46e − 05	0.155278707
(S)‐1‐Hydroxybutyrylcarnitine levels	CXCL1	ieu‐b‐4961	−0.00049044	−4.49e − 05	0.091504112
Ascorbic acid 2‐sulfate levels	AXIN1	ieu‐b‐4961	0.000741968	7.64e − 05	0.102641551
Carotene diol (1) levels	TRANCE	ieu‐b‐4961	0.000500512	1.15e − 05	0.026970684
Aspartate levels	CCL11	ieu‐b‐4961	−0.000529911	−6.42e − 05	0.121124029
*Trans*‐urocanate levels	AXIN1	ieu‐b‐4961	0.000489279	8.52e − 05	0.174096742
X‐18921 levels	AXIN1	ieu‐b‐4961	0.000195602	5.07e − 05	0.128150429
Cysteine‐to‐5‐oxoproline ratio	TRANCE	ieu‐b‐4961	0.000171078	1.27e − 05	0.014026091
Retinol (vitamin A)‐to‐oleoyl‐linoleoyl‐glycerol (18:1–18:2) [2] ratio	IL‐15RA	ieu‐b‐4961	0.000497657	1.51e − 05	0.070926812

### 3.12. Asp Downregulates CCL11 Expression and Secretion in a Dose‐ and Time‐Dependent Manner

To validate the mediation analysis results, in vitro intervention experiments were performed using Asp on the oral squamous cell carcinoma cell line CAL‐27. As shown in Figure 9, CCL11 expression and secretion were significantly downregulated by Asp in both dose‐dependent and time‐dependent manners. At the mRNA level (Figure 9a), the relative expression of CCL11 was shown to gradually decline with increasing Asp concentrations (0–5 mM). This inhibitory effect was most pronounced after 48 h of treatment, where CCL11 mRNA expression was significantly reduced to approximately 0.6‐fold in the 5 mM Asp treatment group compared to the control group (*p* < 0.05). A clear dose–response relationship was demonstrated by the inhibitory effect, with statistically significant differences being observed in both 1 and 5 mM concentration groups compared to the control group (*p* < 0.05). At the protein level (Figure 9b), it was demonstrated by ELISA detection that CCL11 secretion was similarly inhibited by Asp in a dose‐dependent manner. After 24 and 48 h of treatment, CCL11 protein concentrations in cell culture supernatants were observed to decrease significantly with increasing Asp concentrations. After 48 h of treatment, CCL11 protein levels were reduced from approximately 580 pg/mL in the control group to approximately 170 pg/mL in the 5 mM Asp group, representing a reduction of over 70% (*p* < 0.05–0.001).

Figure 9Asp treatment reduces CCL11 expression in CAL‐27 cells. (a) Relative mRNA expression levels of CCL11 in CAL‐27 cells treated with different concentrations of Asp (0, 0.5, 1, and 5 mM) for 6, 12, and 48 h. (b) CCL11 protein concentrations in cell culture supernatants measured by ELISA after 24 and 48 h of Asp treatment at the indicated concentrations. Data are presented as mean ± SEM. Statistical significance:  ^∗^ *p* < 0.05,  ^∗∗^ *p* < 0.01,  ^∗∗∗^ *p* < 0.001, and  ^∗∗∗∗^ *p* < 0.0001.

**Figure:**
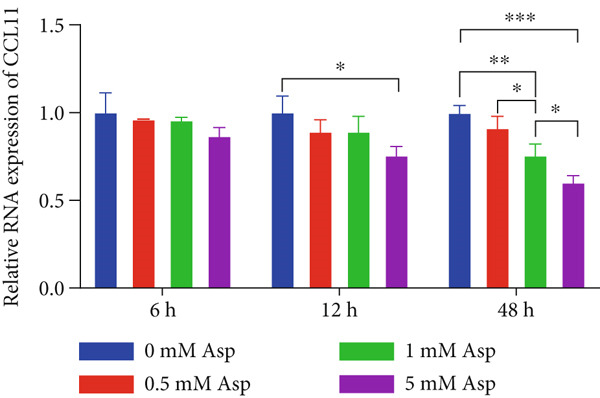
(a)

**Figure:**
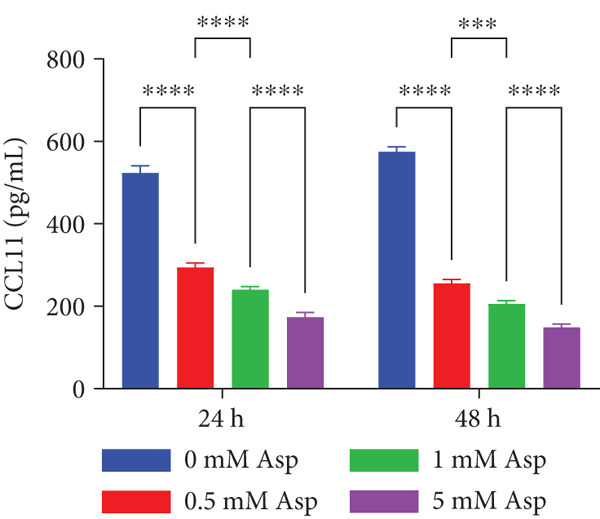
(b)

### 3.13. Asp Suppresses Malignant Phenotypes and Inflammatory Responses in Oral Cancer Cells via Downregulation of CCL11 Expression

CCL11 overexpression markedly enhanced CAL‐27 cell metabolic activity as demonstrated by CCK8 assays (Figure 10a), while Asp treatment substantially reduced cellular metabolic capacity, with partial reversal observed in rescue experiments (*p* < 0.0001). Colony formation assays revealed that CCL11 overexpression significantly promoted clonogenic survival, whereas Asp treatment dramatically suppressed colony formation ability, and Asp partially attenuated CCL11‐mediated clonogenic enhancement (Figure 10b, *p* < 0.001–0.0001). Wound healing assays indicated that CCL11 overexpression accelerated cell migration, while Asp treatment substantially inhibited migratory capacity and moderately counteracted CCL11‐induced migration enhancement (Figure 10c, *p* < 0.01–0.0001). Transwell invasion assays demonstrated that CCL11 overexpression dramatically increased invasive potential, whereas Asp treatment markedly suppressed invasive capacity and partially rescued CCL11‐mediated invasion enhancement (Figure 10d, *p* < 0.0001). qRT‐PCR analysis confirmed that CCL11 overexpression upregulated CCL11 mRNA expression, while Asp treatment downregulated CCL11 expression with partial restoration observed in rescue experiments (Figure 10e, *p* < 0.01–0.001). ELISA analyses revealed that CCL11 overexpression significantly elevated the secretion of CCL11 protein (Figure 10f), IL‐1*β* (Figure 10g), IL‐6 (Figure 10h), and TNF‐*α* (Figure 10i), while Asp treatment substantially reduced all inflammatory cytokine secretions and partially reversed CCL11‐induced cytokine overproduction in rescue experiments (*p* < 0.001–0.0001), collectively establishing that Asp suppresses oral cancer cell malignant behaviors and inflammatory responses through CCL11 downregulation.

Figure 10CCL11 overexpression promotes malignant behaviors and inflammatory responses in oral cancer cells, counteracted by Asp treatment. (a) Cell metabolic activity by CCK8 assay. (b) Colony formation assay with representative images and survival fraction quantification. (c) Wound healing assay with representative images and migration rate analysis. (d) Transwell invasion assay with representative images and invaded cell quantification. (e) CCL11 mRNA expression by qRT‐PCR. Secreted cytokine levels of (f) CCL11, (g) IL‐1*β*, (h) IL‐6, and (i) TNF‐*α* by ELISA. Data represent mean ± SEM from three independent experiments.  ^∗∗^ *p* < 0.01,  ^∗∗∗^ *p* < 0.001, and  ^∗∗∗∗^ *p* < 0.0001.

**Figure:**
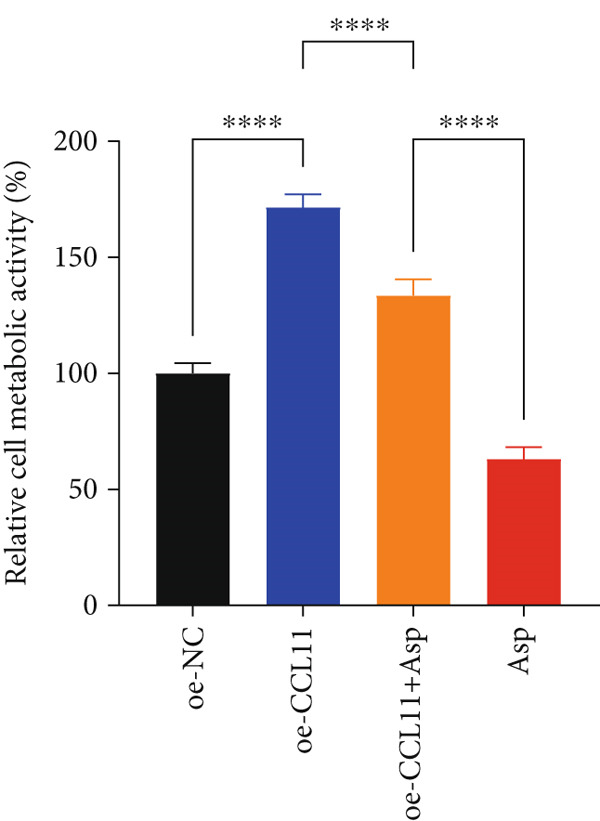
(a)

**Figure:**
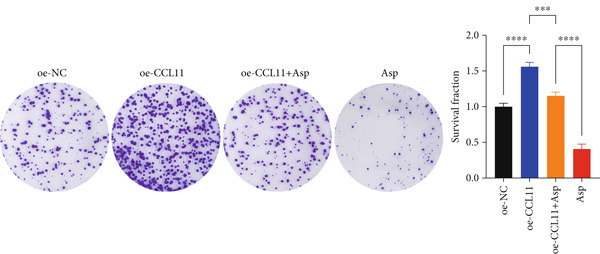
(b)

**Figure:**
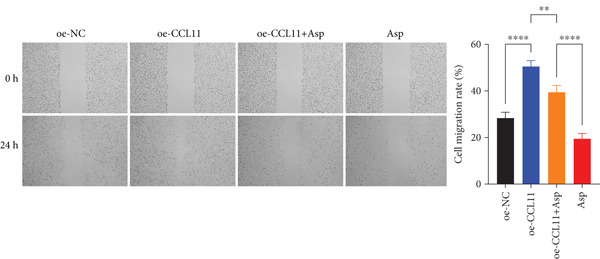
(c)

**Figure:**
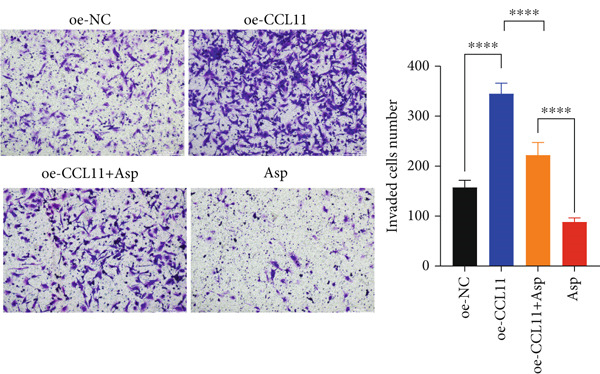
(d)

**Figure:**
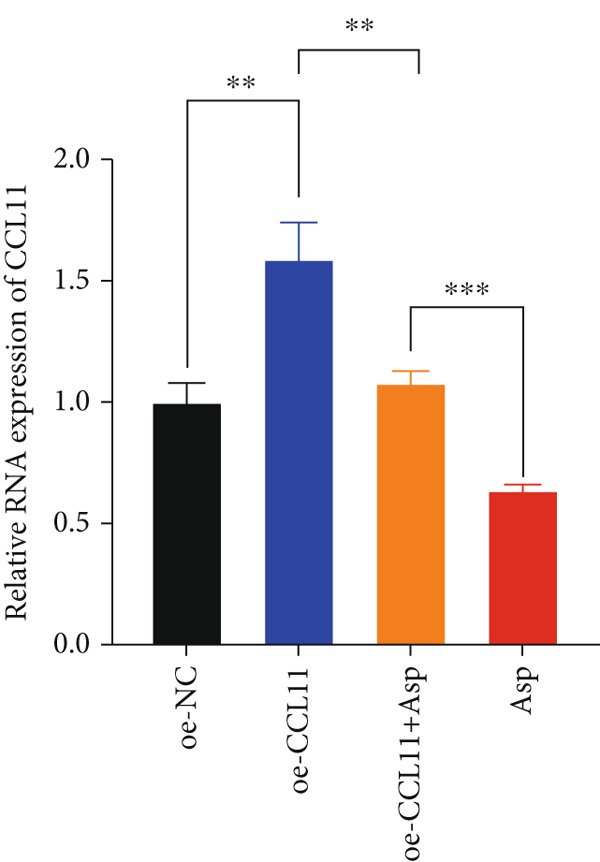
(e)

**Figure:**
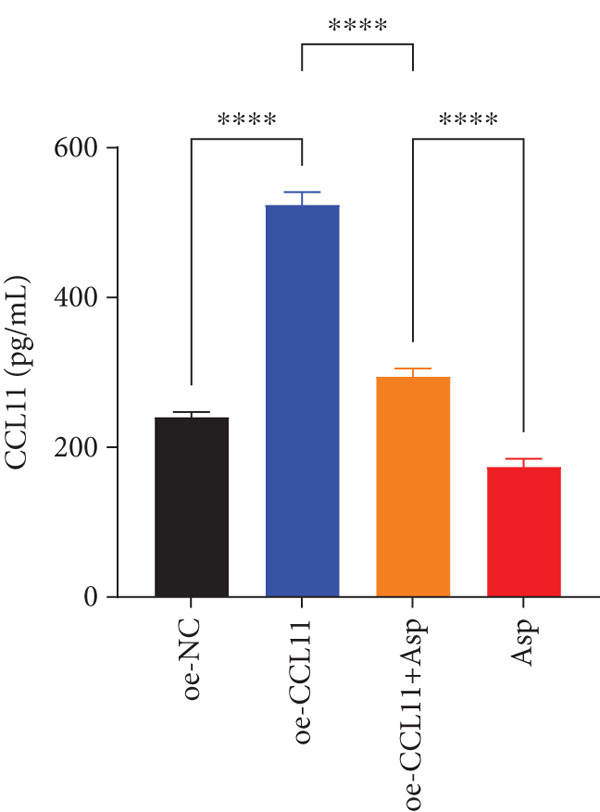
(f)

**Figure:**
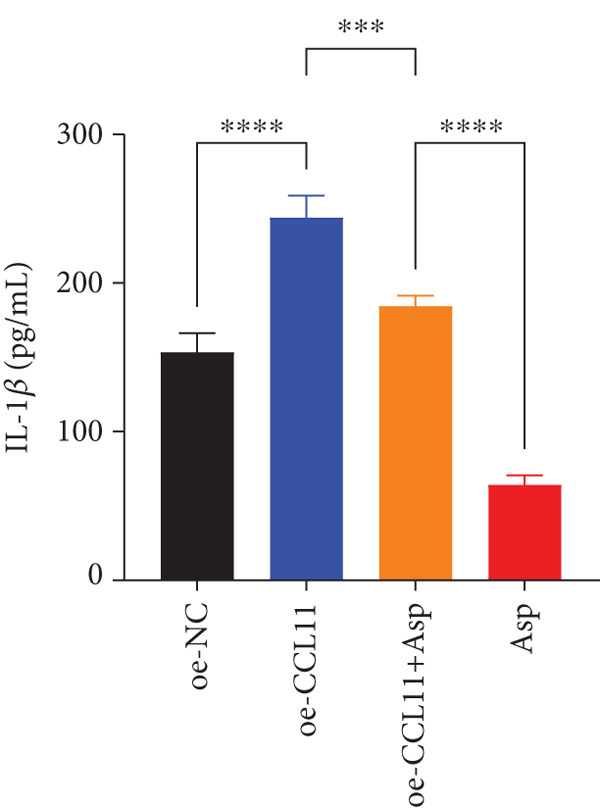
(g)

**Figure:**
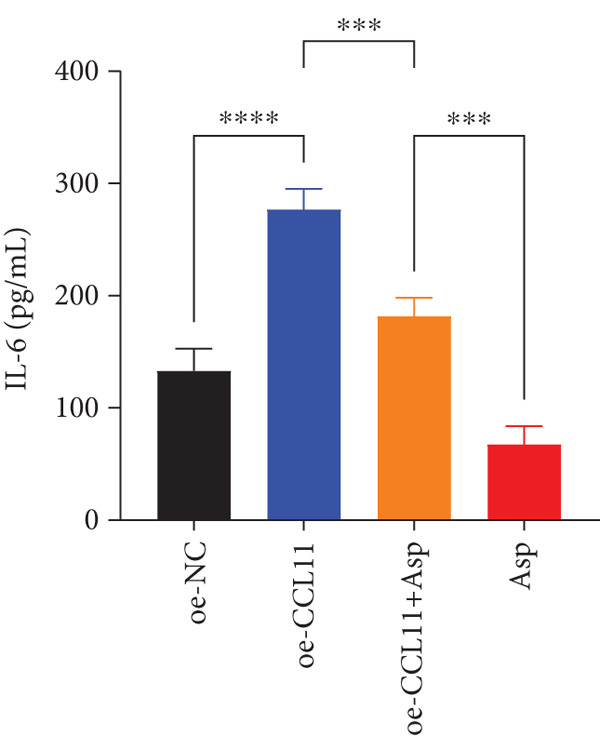
(h)

**Figure:**
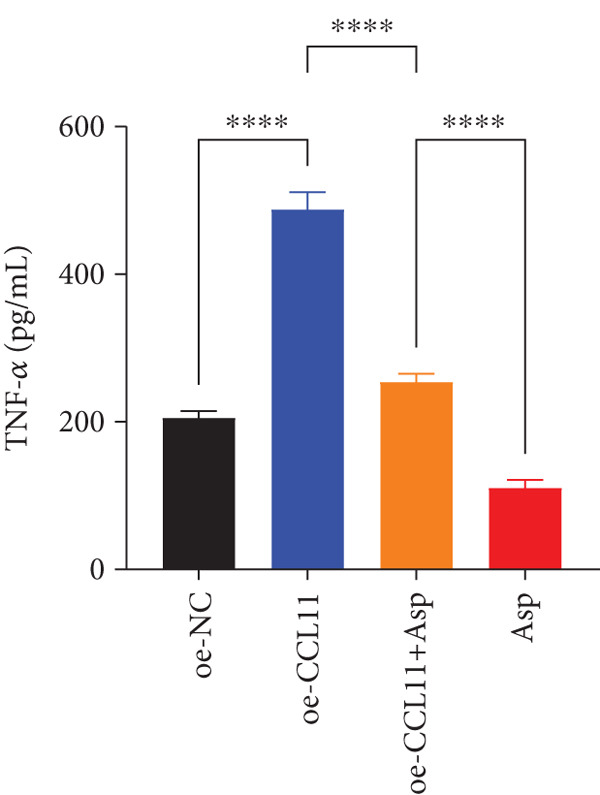
(i)

### 3.14. Asp Regulates CCL11 Expression by Inhibiting NF‐*κ*B and MAPK Signaling Pathway Activation

To elucidate the upstream regulatory mechanisms of CCL11, the activation status of NF‐*κ*B and MAPK signaling pathways was evaluated by detecting the phosphorylation levels of key proteins p65, p38, and ERK. As shown in Figure 11, Western blot analysis revealed the regulatory effects of Asp on key inflammatory signaling pathways. Representative blot images (Figure 11a) demonstrated the protein expression levels of phosphorylated and total forms of NF‐*κ*B p65, MAPK p38, and ERK (1/2) across different experimental groups, with GAPDH serving as the loading control. Quantitative analysis of relative protein expression (Figure 11b) showed that CCL11 overexpression (oe‐CCL11 group) significantly upregulated the expression of phosphorylated NF‐*κ*B p65, MAPK p38, and ERK (1/2) compared to the negative control (oe‐NC group), while Asp treatment (Asp group) markedly suppressed these phosphorylated proteins (*p* < 0.01–0.0001). Furthermore, phosphorylation ratio analysis (Figure 11c) provided more precise measurements of pathway activation, revealing that CCL11 overexpression significantly enhanced the phosphorylation levels of all three key proteins, whereas Asp treatment substantially attenuated these phosphorylation events (*p* < 0.01–0.0001). Notably, cotreatment with Asp effectively reversed the CCL11‐induced upregulation of phosphorylated proteins (oe‐CCL11 + Asp group, *p* < 0.0001), demonstrating that Asp could effectively counteract the CCL11‐mediated activation of these inflammatory signaling cascades.

Figure 11Asp downregulates CCL11 expression via the inhibition of NF‐*κ*B and MAPK signaling pathways. (a) Representative Western blot images of phosphorylated and total NF‐*κ*B p65, MAPK p38, and ERK (1/2) with GAPDH as a loading control. (b) Quantification of relative protein expression levels. (c) Phosphorylation ratios (p‐protein/total protein) indicating pathway activation status. Data represent mean ± SEM from three independent experiments.  ^∗∗^ *p* < 0.01,  ^∗∗∗^ *p* < 0.001, and  ^∗∗∗∗^ *p* < 0.0001; ns, not significant.

**Figure:**
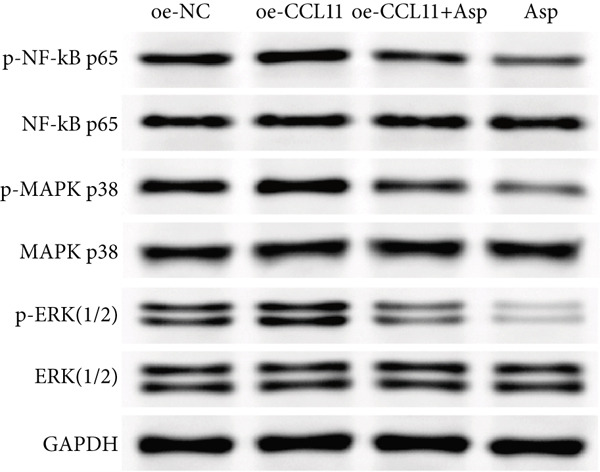
(a)

**Figure:**
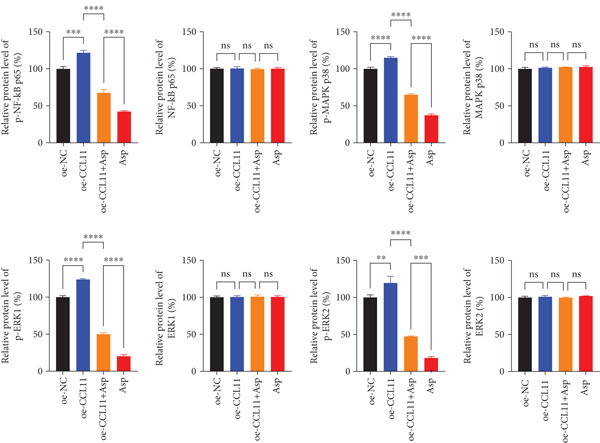
(b)

**Figure:**
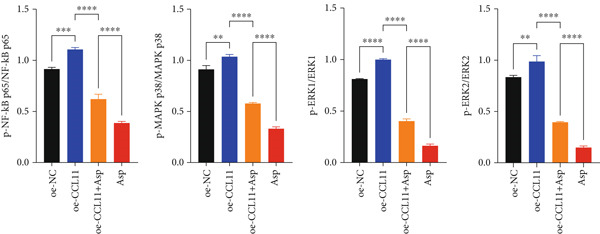
(c)

## 4. Discussion

Our study represents one of the comprehensive applications of MR methodology to systematically investigate the causal relationships between 1400 plasma metabolites, 91 inflammatory factors, and oral cancer risk while simultaneously quantifying mediation effects through inflammatory pathways. While MR studies examining metabolites and cancer risk have been conducted for other cancer types, comprehensive analyses specifically focused on oral cancer using this scale of metabolomic and inflammatory profiling remain limited. This innovative approach addresses critical gaps identified in previous research, which has predominantly relied on observational studies with inherent limitations of confounding and reverse causation. Traditional metabolomic studies in oral cancer have shown promising results for biomarker discovery [32], with researchers identifying distinct metabolic signatures in saliva and tissue samples from oral cancer patients [33]. However, these studies have been limited by their cross‐sectional designs and inability to establish causality. Wei et al. demonstrated that salivary metabolite signatures could distinguish oral cancer from controls [34], while Luo et al. highlighted the potential of metabolomics as diagnostic targets [35]. Our MR framework overcomes these limitations by leveraging genetic variants as instrumental variables, providing robust evidence for causal relationships that cannot be inferred from conventional epidemiological approaches. The systematic review by Pierce et al. emphasized the growing importance of MR in cancer research [36], while Yarmolinsky et al. highlighted its particular promise for identifying interventional targets in cancer prevention [37]. Our comprehensive analysis of 4667 exposure–outcome associations represents the largest systematic evaluation of metabolite–cancer causality to date, significantly expanding upon previous MR studies that typically examined limited numbers of exposures.

Our identification of 61 metabolites with significant causal relationships to oral cancer risk, including 29 protective and 32 risk‐enhancing factors, provides insights into the metabolic underpinnings of oral carcinogenesis. The protective effects of metabolites such as 5,6‐dihydrothymine, 2‐naphthol sulfate, and plasma lactate align with emerging evidence from metabolomic studies suggesting that certain metabolic profiles may confer cancer resistance through enhanced DNA repair mechanisms and cellular homeostasis [38]. Our findings regarding risk‐enhancing metabolites, particularly the carnitine‐to‐acetylcarnitine ratio and various metabolite ratios, corroborate previous observations of altered lipid metabolism in oral cancer pathogenesis [39]. The identification of 14 inflammatory factors with direct causal relationships to oral cancer strongly supports the established paradigm of inflammation‐driven carcinogenesis. Our findings that IL‐8, TNF‐*α*, and IL‐6 serve as risk factors are consistent with extensive literature demonstrating elevated levels of these proinflammatory cytokines in oral cancer patients [40]. The systematic review and meta‐analysis by Huang et al. confirmed TNF‐*α* as having the highest diagnostic accuracy among salivary cytokines [10], while Benito‐Ra et al. emphasized the diagnostic potential of IL‐6 and IL‐8 [41]. Our MR analysis provides causal evidence supporting these associations, indicating that therapeutic strategies targeting these inflammatory pathways could offer preventive potential. The broader cancer literature supports this notion, with chronic inflammation being recognized as a hallmark of cancer development [42] and IL‐6 playing crucial roles in chronic inflammatory diseases and cancer progression [43].

The identification of 15 significant mediation pathways, with mediation ratios ranging from 1.4% to 17.4%, represents a novel contribution to understanding the mechanistic links between metabolic perturbations and oral cancer development. Our finding that *trans*‐urocanate levels demonstrate the highest mediation ratio through AXIN1 (17.4%) provides new insights into how specific metabolites may influence cancer risk through inflammatory mechanisms. This mediation framework is consistent with recent advances in cancer epidemiology that emphasize the importance of understanding intermediate biological pathways [44]. The evidence that IL‐8 and AXIN1 serve as key mediators for multiple metabolites suggests these inflammatory factors function as critical nodes in cancer development pathways, indicating their potential as intervention targets. Large‐scale epidemiological studies have demonstrated associations between various inflammatory markers and cancer incidence across multiple sites [45], with systematic reviews highlighting C‐reactive protein, IL‐6, and TNF‐*α* as consistently associated with increased cancer risk [46]. Recent MR studies have begun to evaluate the causal role of inflammatory markers in cancer risk [47], though none have previously examined the specific context of oral cancer or incorporated comprehensive mediation analysis. Our findings demonstrate that inflammatory pathways mediate a substantial proportion of metabolite effects on cancer risk and offer mechanistic insights that could inform future precision medicine approaches, particularly for identifying high‐risk individuals who may benefit from anti‐inflammatory interventions, though further validation in independent cohorts remains necessary [48].

MR mediation analysis revealed that approximately 12.1% of the total effect of aspartate on oral cancer (beta_all = –0.00052991) was mediated through CCL11 (beta_mediation_ratio = 0.1211), with aspartate significantly reducing CCL11 levels (beta_mediation = –6.42 × 10^−5^) (Table 4), suggesting CCL11 as a critical hub connecting metabolic alterations to inflammation‐driven carcinogenesis. Correspondingly, aspartate significantly inhibited CCL11 expression and secretion in a dose‐ and time‐dependent manner (Figure 9), supporting active metabolite regulation of inflammatory signaling [49]. Validation experiments demonstrated that CCL11 overexpression promoted oral squamous cell carcinoma cell proliferation, colony formation, migration, invasion, and upregulation of key inflammatory cytokines (IL‐1*β*, IL‐6, and TNF‐*α*) (Figure 10), consistent with its protumorigenic properties [50], while these effects were effectively reversed by aspartate. Mechanistically, aspartate significantly reduced phosphorylation activation of upstream NF‐*κ*B and MAPK (p38 and ERK) pathways regulating CCL11 expression [51, 52], thereby downregulating CCL11 and blocking subsequent inflammatory and procarcinogenic cascades (Figure 11). Collectively, this study establishes a complete aspartate–NF‐*κ*B/MAPK–CCL11–inflammation–oral cancer evidence chain from genetic causal inference to molecular mechanism validation, providing novel mechanistic foundations for metabolism–inflammation targeted interventions.

Although our MR–Egger pleiotropy tests revealed no evidence of significant horizontal pleiotropy (all *p* values > 0.05, Table 2) and heterogeneity analyses demonstrated consistent effect estimates across genetic instruments (all *Q*_*p*val > 0.05, Table 1), we acknowledge inherent limitations in pleiotropy detection methodologies. While large‐scale analyses examining 1400 metabolites with thousands of genetic variants may theoretically enhance the probability of undetected horizontal pleiotropy [53], our rigorous multifaceted sensitivity analysis framework provides substantial evidence for the robustness of our findings. Although previous large‐scale MR investigations have documented the potential presence of horizontal pleiotropy in complex trait analyses [53], we mitigated pleiotropic bias through the implementation of multiple complementary MR methodologies, stringent instrumental variable selection criteria, and comprehensive sensitivity assessments. While contemporary pleiotropy detection methods possess inherent limitations in identifying certain forms of balanced pleiotropy [16], the concordance across all sensitivity analyses, stability demonstrated in leave‐one‐out evaluations, and statistical significance of Steiger directionality tests collectively substantiate the validity of our identified causal relationships. Moreover, the biological plausibility of the metabolite–oral cancer associations we characterized further reinforces the credibility and interpretability of our results.

While our study has certain inherent limitations, such as population representativeness and sample size constraints, these also provide important opportunities for future research development. With the rapid advancement of multiomics technologies and the establishment of large‐scale biobanks, future studies are expected to validate our findings in larger, more diverse populations and explore causal relationships across different genetic backgrounds and environmental contexts [54]. The emergence of molecular subtyping and precision medicine in oral cancer offers new perspectives for understanding disease heterogeneity, with the metabolic and inflammatory biomarkers we identified holding promise for establishing the foundation for future development of personalized risk assessment models [55]. While the integration of artificial intelligence and machine learning technologies may facilitate biomarker discovery and screening, their clinical applicability in oral cancer diagnosis necessitates systematic validation through independent prospective cohorts to evaluate real‐world diagnostic accuracy and stability [56]. They offer new intervention directions for oral cancer prevention and treatment. Emerging biosensor technologies and liquid biopsy methods provide technical platforms for exploring the clinical translation of research findings [57]. While multidisciplinary collaborative research integrating metabolomics, inflammatory biology, genetics, and clinical medicine may enhance our understanding of oral cancer pathogenesis, the clinical translation of these findings necessitates rigorous validation through multicenter prospective studies [54]. With the continued decline in sequencing technology costs and enhanced bioinformatics analysis capabilities, large‐scale prospective cohort studies will become crucial platforms for validating causal relationships and developing prevention strategies. The causal inference evidence provided by this study holds promise for supporting the transformation of oral cancer management paradigms, promoting a shift from the traditional “treatment‐focused” approach toward a “prevention‐first, precision intervention” model, although this transformation requires additional validation studies and clinical evidence [58].

## 5. Conclusions

This MR study identified 61 metabolites and 14 inflammatory factors with significant causal associations with oral cancer risk while revealing 15 mediation pathways (mediation proportions ranging from 1.4% to 17.4%). The aforementioned causal inferences were based on genome‐wide SNPs as instrumental variables, reflecting the effects of natural germline genetic variations on metabolic and inflammatory characteristics. Cellular experimental validation of the predicted “aspartate–CCL11–oral cancer” pathway further demonstrated that aspartate can dose‐dependently suppress CCL11 expression and its driven malignant phenotypes, primarily through the inhibition of NF‐*κ*B and MAPK signaling pathways and the attenuation of inflammatory responses. The high concordance between computational results and experimental evidence supports the existence of a “metabolism–inflammation–cancer” axis and suggests that relevant molecules in this pathway may serve as potential intervention targets for precision prevention and treatment of oral cancer.

## Ethics Statement

The authors have nothing to report.

## Disclosure

All authors provided critical feedback and helped shape the research, analysis, and manuscript. All data were generated in‐house, and no paper mill was used. All authors agree to be accountable for all aspects of work, ensuring integrity and accuracy. The authors take full responsibility for the final content.

## Conflicts of Interest

The authors declare no conflicts of interest.

## Author Contributions

Shaonan Hu: funding acquisition, methodology, investigation, formal analysis, data curation, writing—original draft, writing—review and editing, and project administration. Chufeng Liu: project administration, supervision, funding acquisition, resources, and conceptualization. Shaonan Hu and Chufeng Liu contributed equally as the corresponding authors to this work.

## Funding

This study was funded by the Science Research Cultivation Program of Stomatological Hospital, Southern Medical University (PY2024006).

## Data Availability

The data used and analyzed during the current study are available from the corresponding author upon reasonable request.
